# 

**Published:** 1921-08-06

**Authors:** 


August 6, 1921] [Price Twopence

### The Hospital

### The Workers' Newspaper of Administrative Medicine and Institutional Life, Administration, National Insurance and Health.

CONTENTS

**Doctors' Fees and Patients' Payments** ... ... 309

**The New Local Hospital Committees** ... ... 310

**Bradford Hospital Fund** ... ... ... ... 310

**Notes of the Week** ... ... ... ... ... ... 311

Ideas for Hospital Week—The Poor Law and the Voluntary Hospitals Commission—The First-Aid Services—Cancer Research—For Service to Humanity—The "Japan Medical World"—The "Journal of Obstetrics and Gynæcology"—Admission of Accident Cases—An Example to Birmingham—The Sheffield Contribution Scheme—Spirit Duty (Voluntary Hospitals) Grant.

**A Public Safeguard** ... ... ... ... ... 313

Work of the Port of London Sanitary Committee.

**Literature and License...** ... ... ... ... ... 314

**The Hospital Commissariat** ... ... ... ... 315

Buying Difficulties of the Present Day.

**Rate-Provided Hospitals** ... ... ... ... ... 316

The Relation of the Medical Profession.

**The Public Well-Being** ... ... ... ... ... 317

The Westminster Conference—A Complete Programme of Child Welfare.

**Parliament and the Professions** ... ... ... 318

**Maternity Home Supervision** ... ... ... 318

**Metropolitan Hospital Sunday Fund** ... ... 319

Distribution of £100,170.

**General Nursing Council for Scotland** ... ... 320

**View Day at the Great Northern Hospital** ... 320

**The Matrons' and Sisters' Department** ... ... 321

The Uses of a Syllabus.

**Round the Hospitals** ... ... ... ... ... ... 321

**The State Registers** ... ... ... ... ... ... vi

The Minimum Qualifications for Admission.

**The Scottish Syllabus** ... ... ... ... ... viii

**The Tuberculosis Conference...** ... ... ... ... viii

